# Sirtuin 5 levels are limiting in preserving cardiac function and suppressing fibrosis in response to pressure overload

**DOI:** 10.1038/s41598-022-16506-7

**Published:** 2022-07-18

**Authors:** Angela H. Guo, Rachael Baliira, Mary E. Skinner, Surinder Kumar, Anthony Andren, Li Zhang, Robert S. Goldsmith, Shaday Michan, Norma J. Davis, Merissa W. Maccani, Sharlene M. Day, David A. Sinclair, Matthew J. Brody, Costas A. Lyssiotis, Adam B. Stein, David B. Lombard

**Affiliations:** 1 Department of Pathology, University of Michigan, Ann Arbor, MI 48109 USA; 2 Molecular and Cellular Pathology Graduate Program, University of Michigan, Ann Arbor, MI 48109 USA; 3 Department of Molecular and Integrative Physiology, University of Michigan, Ann Arbor, MI 48109 USA; 4 Department of Pharmacology, University of Michigan, Ann Arbor, MI 48109 USA; 5 Pharmacology Graduate Program, University of Michigan, Ann Arbor, MI 48109 USA; 6 Department of Genetics, Harvard Medical School, Boston, MA 02115 USA; 7 Rejuvenate Bio Inc, San Diego, CA 92121 USA; 8 Division of Cardiovascular Medicine, University of Pennsylvania, Philadelphia, PA USA; 9 Department of Internal Medicine, University of Michigan, Ann Arbor, MI 48109 USA; 10 Rogel Cancer Center, University of Michigan, Ann Arbor, MI 48109 USA; 11 Institute of Gerontology, University of Michigan, Ann Arbor, MI 48109 USA; 12 Department of Pathology and Laboratory Medicine, University of Miami Miller School of Medicine, 708 Biomedical Research Building, 1501 NW 10th Avenue, Miami, FL 33136 USA; 13 Sylvester Comprehensive Cancer Center, Miami, FL 33136 USA

**Keywords:** Metabolomics, Cardiac hypertrophy, Post-translational modifications, Transcriptomics

## Abstract

Heart failure (HF) is the inability of the heart to pump blood sufficiently to meet the metabolic demands of the body. HF with reduced systolic function is characterized by cardiac hypertrophy, ventricular fibrosis and remodeling, and decreased cardiac contractility, leading to cardiac functional impairment and death. Transverse aortic constriction (TAC) is a well-established model for inducing hypertrophy and HF in rodents. Mice globally deficient in sirtuin 5 (SIRT5), a NAD^+^-dependent deacylase, are hypersensitive to cardiac stress and display increased mortality after TAC. Prior studies assessing SIRT5 functions in the heart have all employed loss-of-function approaches. In this study, we generated SIRT5 overexpressing (SIRT5OE) mice, and evaluated their response to chronic pressure overload using TAC. Compared to littermate controls, SIRT5OE mice were protected against adverse functional consequences of TAC, left ventricular dilation and impaired ejection fraction. Transcriptomic analysis revealed that SIRT5 suppresses key HF sequelae, including the metabolic switch from fatty acid oxidation to glycolysis, immune activation, and fibrotic signaling pathways. We conclude that SIRT5 is a limiting factor in the preservation of cardiac function in response to experimental pressure overload.

## Introduction

Heart failure (HF) represents the end stage of a variety of cardiovascular pathologies, leading to insufficient oxygen delivery to meet the metabolic demands of the body. The incidence of HF is predicted to increase by 46% between 2012 and 2030 in the US, mainly due to an increase in population longevity coupled with improved survival rates following cardiovascular injury^1^. In 2018 alone, HF claimed 83,616 lives^2^. HF with systolic dysfunction is characterized by a progressive decline in contractile function and chronic hemodynamic overload, and by ventricular hypertrophy and remodeling, neurohormonal compensation mechanisms, and myocardial damage. Key cellular mechanisms thought to contribute to HF include myocyte death, reduced ability to maintain calcium homeostasis, and changes in the production and utilization of high-energy phosphates^3^. However, the molecular changes that lead to HF are still incompletely understood, and currently there are no mechanism-based treatments for HF.

Transverse aortic constriction (TAC) is a well-established experimental rodent model for pressure overload-induced HF. In TAC, the aorta is surgically narrowed, chronically increasing resistance to outflow from the left ventricle (LV)^4^. The resulting increased left ventricular burden initially induces concentric hypertrophy to compensate for TAC-induced elevated resistance. However, chronic pressure overload leads to contractile dysfunction and extensive remodeling, including fibrosis and ventricular dilation, resulting in functional decline. Over time, the deteriorating LV becomes unable to meet the hemodynamic needs of the body, resulting in progressive HF^5^.

Sirtuins are a family of NAD^+^-dependent deacylases playing major roles in cellular stress responses. In mammals, each of the seven sirtuins possesses a distinct combination of enzymatic activities, molecular targets, subcellular localization, and tissue expression^6^. Sirtuin 5 (SIRT5) possesses robust deacylase activity against lysine succinylation, malonylation, and glutarylation, negatively-charged lysine acyl modifications derived from their respective Coenzyme A (CoA) species^7–11^. SIRT5 target proteins reside in the mitochondrial matrix, cytosol, peroxisome, and nucleus; many function in metabolic pathways, including the citric acid cycle: *e.g.* pyruvate dehydrogenase and succinate dehydrogenase (SDH), and fatty acid oxidation: *e.g.* trifunctional enzyme subunit alpha (ECHA)^8,12^. Although many SIRT5 targets take part in key metabolic pathways, no striking metabolic phenotypes have been observed in *Sirt5* knockout (SIRT5KO) or overexpressing (OE) mice under basal, unstressed conditions^13–15^.

Among normal tissues, a major site of SIRT5 function appears to be the heart. SIRT5KO mice display a modest, age-associated decrease in cardiac function, along with increased fibrosis and hypertrophy^12^. SIRT5KO mice are also hypersensitive to cardiac stress. In an ischemia–reperfusion (I/R) injury model, SIRT5KO mice show increased infarct size and impaired recovery^16^. In these studies, proteomic experiments identified ECHA and SDH, respectively, as the likely substrates most relevant to SIRT5’s cardioprotective activity. In response to TAC, whole-body SIRT5KOs developed more severe cardiac dysfunction, exacerbated hypertrophy, and increased fibrosis compared to wild-type (WT) littermates^17^. The authors of this study proposed that, during TAC, SIRT5 deficiency is associated with impaired electron flow in oxidative phosphorylation, elevated mitochondrial NADH levels, and impaired oxidative metabolism, with greater reliance on glycolysis. However, a subsequent study by the same group using a cardiomyocyte (CM)-specific SIRT5KO strain showed no differences between WT and SIRT5 mutants in response to TAC^18^.

In this study, we generated SIRT5 overexpressing (SIRT5OE) mice and evaluated their response to cardiac pressure overload. SIRT5OE mice displayed no obvious cardiac phenotype and very few gene expression changes in the absence of stress compared to WT littermates. However, SIRT5OE mice showed robust protection against TAC-induced LV dilation and subsequent functional decline. Transcriptomic analysis linked the protection conferred by SIRT5 overexpression to suppression of key transcriptional and signaling events accompanying ventricular hypertrophy and HF, including cardiac fibrosis, inflammatory cytokine signaling, and the metabolic switch from fatty acid oxidation to glycolysis. Our results suggest that SIRT5 levels are limiting in the context of the cardiac response to pressure overload.

## Results

### Generation and characterization of SIRT5-overexpressing mice

**Figure 1:**
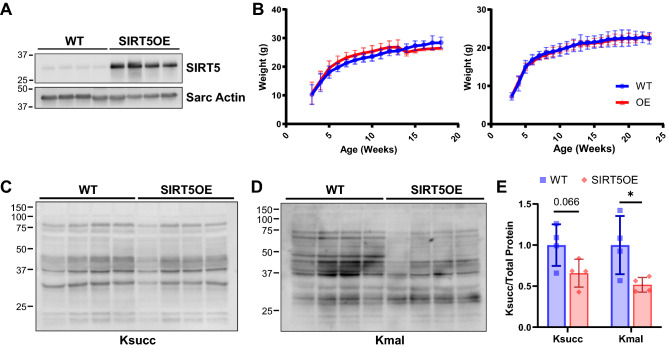
SIRT5OE mice exhibit decreased mildly decreased cardiac lysine malonylation. **(A)** Immunoblot analysis of SIRT5 expression in heart samples presented in c-e. Original immunoblot images are presented in Supplemental Fig. 7A. **(B)** Weight (g) of WT and SIRT5OE male and female mice with age. **(C-E)** Representative immunoblot analysis of succinyl-lysine (Ksucc), and malonyl-lysine (Kmal) levels in WT and SIRTOE hearts, with quantification. Original immunoblot images are presented in Supplemental Fig. 7B-C.

To investigate the effects of SIRT5 on cardiac function, we generated a transgenic SIRT5 overexpressing (SIRT5OE) mouse strain by inserting a LoxP-STOP-LoxP-SIRT5 cassette into the 3’UTR of the *Col1A1* locus, using a well-characterized transgene insertion system (Fig. 1A)^19^. Constitutive, whole-body SIRT5 overexpression is driven by the CAGGS promoter (Supplemental Fig. 1A)^20^. SIRT5OE mice were born at normal Mendelian and sex ratios, and grossly indistinguishable from their WT littermates, with no obvious differences in weight gain with age in either sex (Supplemental Fig. 1B, Fig. 1B). We tested the molecular effects of SIRT5 overexpression on bulk protein acylation in the heart and found modest decreases in total lysine succinylation (Ksucc) and lysine malonylation (Kmal), which were significant for Kmal (Fig. 1C-E).

### SIRT5 overexpression protects against TAC-induced heart failure

**Figure 2:**
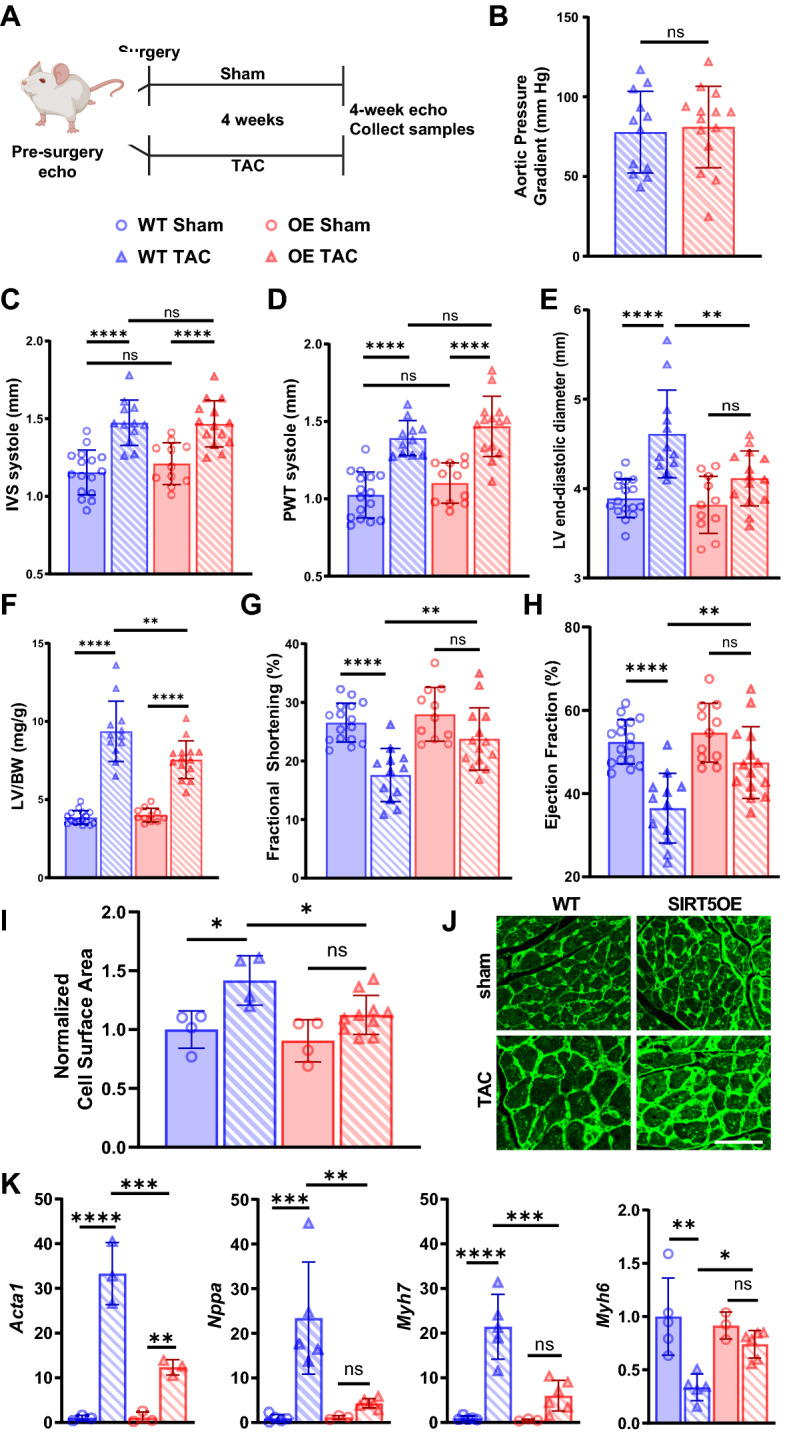
SIRT5OE mice are protected against TAC-induced heart failure. Echocardiography was performed on WT sham (*n* = 16), WT TAC (*n* = 12), SIRT5OE sham (*n* = 10) and SIRT5OE TAC (n = 14) mice to measure changes in cardiac function four weeks post-surgery. **(A)** Depiction of groups, procedures, and timeline of surgery. **(B)** Aortic pressure gradient in mice after TAC. Echo measurements for **(C)** systolic interventricular septum (IVS) thickness; **(D)** systolic posterior wall thickness (PWT); **(E)** LV end-diastolic diameter; **(F)** left ventricle mass normalized to body weight; **(G)** fractional shortening; **(H)** ejection fraction. **(I)** Quantification of CM cell area, normalized to WT sham four weeks after surgery [WT sham (*n* = 4), WT TAC (*n* = 4), SIRT5OE sham (*n* = 4) and SIRT5OE TAC (*n* = 10)]. **(J)** Representative wheat germ agglutinin-stained cardiac sections of the indicated genotypes and treatments four weeks post-surgery. Scale bar = 50 um. **(K)** qRT-PCR for *Acta1* (n = 3 for all groups); *Nppa* and *Myh6* [WT sham (*n* = 5), WT TAC (*n* = 5), SIRT5OE sham (*n* = 3) and SIRT5OE TAC (*n* = 6)]; and *Myh7* [WT sham (*n* = 5), WT TAC (*n* = 4), SIRT5OE sham (*n* = 3) and SIRT5OE TAC (*n* = 5)] expression normalized to GAPDH. Statistical significance was determined using Student’s t-test for 2-group analysis or two-way ANOVA followed by Sidak’s correction for multiple comparisons for 4-group analyses.

To evaluate sensitivity to cardiac stress, male WT and SIRT5OE littermates between 4–8 months of age were randomly assigned to a sham or TAC operation (Fig. 2A)^21^. TAC surgically narrows the transverse aorta, generating a pressure gradient that reflects the severity of pressure overload on the heart. Sham animals undergo an identical surgical procedure but without aortic banding. Echocardiograms (echos) to assess cardiac structure and function were performed prior to surgery, and then repeated at 4-weeks, at which point tissue was collected for downstream analyses (Fig. 2A). WT and SIRT5OE sham animals did not exhibit any notable differences by echo, indicating that global SIRT5 overexpression does not alter cardiac structure or function under basal conditions (Table 1). Additionally, the magnitude of the aortic pressure gradient was comparable between WT and SIRT5OE TAC mice (Fig. 2B). Thus, any differences observed between genotypes following TAC were not due to discrepancies in the degree of cardiac outflow obstruction. Pressure overload first induces concentric cardiac hypertrophy, a temporary compensatory mechanism to relieve stress on the heart^22^. Echo-based measurement of the interventricular septum (IVS) and the posterior wall (PW) thickness during both systole and diastole showed comparable LV hypertrophy at four weeks after TAC in all mice irrespective of genotype (Fig. 2C-D, Table 1). Consistent with other studies, the TAC procedure itself was associated with a 15–18% mortality rate, with 3 mice and 4 mice dying in the WT and SIRT5OE groups respectively (Supplemental Table 1). Therefore, SIRT5 overexpression does not affect cardiac concentric hypertrophy in response to TAC.

**Table 1:** Echocardiogram measurements 4 weeks after surgery in WT, SIRT5 sham and TAC mice.

	WT sham (*n* = 16)	WT TAC (*n* = 12)	SIRT5OE sham (*n* = 11)	SIRT5OE TAC (*n* = 14)	Genotype Effect	TAC Effect	Interaction Effect
Age at Procedure (months)	5.32 ± 0.60	5.26 ± 0.50	5.88 ± 0.82	5.26 ± 0.46	0.6473	0.5728	0.6469
Heart rate (bpm)	504.75 ± 13.31	487.17 ± 19.47	502.26 ± 17.59	452.04 ± 14.58	0.250	0.041*	0.317
LV Mass/BW	3.86 ± 0.11	9.37 ± 0.56	4.01 ± 0.13	7.55 ± 0.32	0.012*	< 0.001*	0.003*
IVS thickness diastole (mm)	0.77 ± 0.02	1.18 ± 0.03	0.80 ± 0.03	1.15 ± 0.03	0.937	< 0.001*	0.217
IVS thickness systole (mm)	1.15 ± 0.04	1.47 ± 0.04	1.21 ± 0.04	1.47 ± 0.04	0.540	< 0.001*	0.433
LV diameter diastole (mm)	3.89 ± 0.05	4.61 ± 0.14	3.82 ± 0.10	4.11 ± 0.08	0.004*	< 0.001*	0.027*
LV diameter systole (mm)	2.86 ± 0.05	3.81 ± 0.15	2.72 ± 0.10	3.14 ± 0.09	< 0.001*	< 0.001*	0.007*
PW thickness diastole (mm)	0.72 ± 0.02	1.12 ± 0.04	0.78 ± 0.03	1.14 ± 0.04	0.208	< 0.001*	0.523
PW thickness systole (mm)	1.02 ± 0.04	1.35 ± 0.05	1.10 ± 0.04	1.47 ± 0.05	0.082^+^	< 0.001*	0.993
LV Volume diastole (uL)	65.76 ± 2.11	99.41 ± 7.53	63.33 ± 3.74	75.44 ± 3.51	0.004*	< 0.001*	0.018*
LV Volume systole (uL)	31.35 ± 1.39	63.99 ± 6.57	29.05 ± 2.65	39.86 ± 2.70	< 0.001*	< 0.001*	0.004*
Ejection Fraction (%)	0.52 ± 0.01	0.36 ± 0.02	0.55 ± 0.02	0.47 ± 0.02	0.002*	< 0.001*	0.036*
Fractional Shortening (%)	0.27 ± 0.01	0.18 ± 0.01	0.28 ± 0.02	0.24 ± 0.01	0.003*	< 0.001*	0.061^+^

Values listed as mean ± standard error of the mean (SEM). Statistical significance was determined using two-way ANOVA. Significance markers: (*) *p* < 0.05, ( +) *p* < 0.1

After prolonged pressure overload, concentric hypertrophy progresses to ventricular dilation and heart failure (HF)^5^. Four weeks after TAC, WT mice showed significantly increased LV diameter, indicating a transition from adaptive to maladaptive ventricular hypertrophy. In comparison, LV diameter did not significantly increase in SIRT5OE TAC mice (Fig. 2E, Table 1). The combination of ventricular hypertrophy and dilation was also reflected in the LV size normalized to body weight. Both genotypes showed increased normalized LV mass after TAC; however, this increase was blunted by SIRT5 overexpression (Fig. 2F). Ventricular dilation leads to reduced fractional shortening (FS) and impaired ejection fraction (EF), a measure of systolic function. Both were significantly reduced in response to chronic pressure overload in the WT mice but preserved in the SIRT5OE mice (Fig. 2G-H). These patterns were also reflected in CM size, with WT TAC mice showing a significant increase in CM area compared to both WT sham and SIRT5OE TAC groups (Fig. 2I-J).

To complement echo-based measurements, we assessed RNA levels of standard markers of cardiac hypertrophy and HF, skeletal muscle Actin (*Acta1*) and atrial natriuretic peptide (*Nppa*)^23^. As expected, expression of both genes increased in response to TAC, but this increase was blunted in SIRT5OE TAC mice compared to WT TAC (Fig. 2K). The failing myocardium also shifts contractile protein expression from myosin heavy chain isoform α (*Myh6*) to β (*Myh7*) (Fig. 2K)^23^. The decrease in *Myh6* expression by TAC was attenuated in SIRT5OE mice compared to WT controls. Overall, based on these physiological and molecular assays, we conclude that SIRT5 overexpression protects against the transition from adaptive, concentric hypertrophy to maladaptive ventricular dilation and systolic dysfunction.

### SIRT5 overexpression mitigates transcriptomic changes induced by TAC

**Figure 3:**
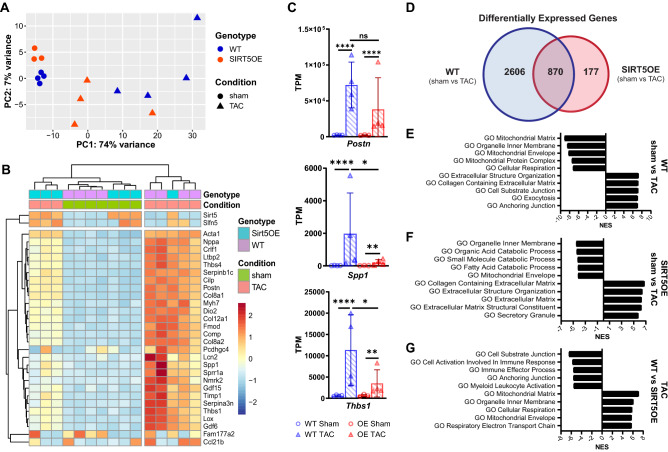
Transcriptomic analysis of heart tissue four weeks after TAC identifies a dampened transcriptional response to pressure overload in SIRT5OE mice. **(A)** Principal component analysis (PCA) of RNA-sequencing data of the four groups of mice [WT sham (*n* = 4), WT TAC (*n* = 4), SIRT5OE sham (*n* = 3) and SIRT5OE TAC (*n* = 4)]. **(B)** Hierarchical clustering analysis (HCA) of the RNA-seq data based on the top 30 genes, determined by greatest variance from the mean. Genes with higher expression compared to the mean skew red; genes with lower expression compared to the mean skew blue. **(C)** *Postn*, *Spp1*, and *Thbs1* mRNA levels measured by RNA-seq. **(D)** Venn Diagram comparing exclusive and overlapping differentially expressed genes in the WT (sham vs TAC) mice and the SIRT5OE (sham vs TAC) mice. **(E–G)** Top five positively and negatively enriched Gene Ontology (GO) pathways from the indicated comparisons, determined by gene set enrichment analysis (GSEA). NES: normalized enrichment score. All listed pathways were significantly enriched with a false discovery rate (FDR) < 0.0001.

To understand how SIRT5 overexpression protects against TAC-induced stress, we performed RNA-seq transcriptomic profiling on whole hearts from WT sham, WT TAC, SIRT5OE sham, and SIRT5OE TAC mice four weeks after surgery. Principal component analysis (PCA) showed that SIRT5 overexpression did not appreciably alter baseline gene expression in the heart, as sham mice clustered together regardless of genotype. In contrast, TAC induced a marked transcriptional response in both genotypes. Apart from one animal, SIRT5OE TAC mice clustered between sham animals and WT TAC animals, suggesting that SIRT5OE blunts the overall TAC transcriptional response (Fig. 3A). Hierarchical cluster analysis (HCA) of the top 30 genes ranked by variance generated a dendrogram with two major clades (Fig. 3B). *Sirt5* transcript levels were elevated in the SIRT5OE samples but were not altered by TAC in either genotype (Fig. 3A, Supplemental Fig. 2A-B). Apart from one animal, all SIRT5OE mice clustered together with WT sham mice in one clade. The second clade contained all WT TAC mice and one SIRT5OE TAC animal. Genes in signaling pathways important to heart failure were evident in this unbiased HCA clustering (Fig. 3B). Consistent with the qRT-PCR data (Fig. 2K), atrial natriuretic peptide (*Nppa*), skeletal muscle Actin (*Acta1*), and myosin heavy chain isoform α (*Myh6*) increased with TAC but these increases were blunted in SIRT5OE TAC mice compared to WT TAC mice (Fig. 3B). Fibroblast remodeling of the extracellular matrix (ECM) and increased fibrosis is a key component of maladaptive remodeling in HF. *Postn*, a key marker of cardiac fibroblast activation and differentiation, was strongly induced in WT TAC samples, but trended less so in the SIRT5OE TAC mice (Fig. 3C)^24,25^. Markers of monocyte-derived macrophages that stimulate fibrosis, *Spp1* and *Thbs1*, were also elevated in TAC samples, and this increase was significantly mitigated in the SIRT5OE mice (Fig. 3C)^26^.Thus, based on both PCA and HCA, SIRT5OE TAC mice are generally more transcriptionally similar to WT sham mice than WT TAC mice.

We defined the criteria for differentially expressed genes (DEGs) between two groups as a false discovery rate (FDR) < 0.05 and fold-change of |1|. Strikingly, there were only 8 DEGs, including SIRT5, between sham WT and SIRT5OE mice, consistent with the observation that SIRT5 overexpression does not significantly alter baseline cardiac parameters. Following TAC, the number of DEGs increased dramatically in both genotypes. There were 2,606 unique DEGs between WT sham and TAC hearts, but only 177 in SIRT5OE sham and SIRT5OE TAC. There were 870 DEGs in common between the WT and SIRT5OE sham to TAC comparisons, representing a core group of genes that respond to chronic pressure overload across both genotypes (Fig. 3D). The lower number of DEGs in SIRT5OE mice after TAC suggests that transcriptional responses induced by pressure overload are partially mitigated by SIRT5 overexpression.

Unbiased analysis of gene signatures enriched by the DEGs of each group was performed using Ingenuity Pathway Analysis (IPA) and Gene Set Enrichment Analysis (GSEA)^27,28^. The top 5 most positively and negatively enriched Gene Ontology (GO) pathways based on normalized enrichment score (NES) were extracellular matrix (ECM) remodeling and metabolic pathways, respectively in sham to TAC comparisons of both genotypes, albeit to different magnitudes (Fig. 3E-F). The most enriched GO terms between TAC samples revealed that SIRT5OE TAC hearts show reduced activation of the immune system and suppressed ECM organization while maintaining expression of respiratory genes compared to WT TAC hearts (Fig. 3G). IPA analysis of these samples showed similar patterns of pathway enrichment (Supplemental Fig. 2C-E). In summary, based on PCA, HCA, and function enrichment analysis, transcriptomic analysis supports the conclusion that SIRT5 overexpression protects against pressure overload-induced HF partially through the suppression of molecular signaling pathways responsible for HF progression.

### Pressure overload induces similar metabolic changes in both WT and SIRT5OE hearts

**Figure 4:**
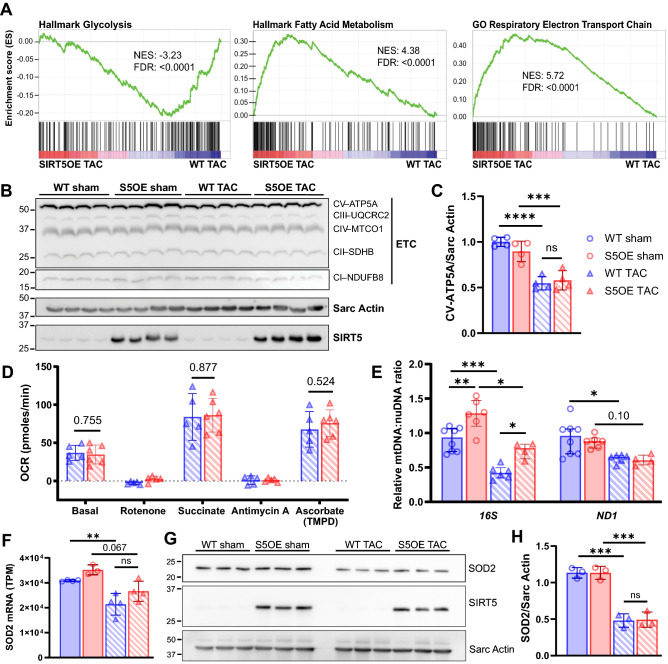
WT TAC mice exhibit a metabolic shift by RNA-seq, but are functionally comparable to SIRT5OE TAC mice four weeks post-surgery. **(A)** GSEA of SIRT5OE TAC compared to WT TAC RNA-seq samples. NES: normalized enrichment score. FDR: false discovery rate. **(B-C)** Immunoblot analysis of electron transport chain subunits and quantification of CV-ATP5A. Original immunoblot images are presented in Supplemental Fig. 8A. **(D)** Agilent/Seahorse analysis of electron flow through the ETC in mitochondria isolated from WT (*n* = 5) and SIRT5OE (*n* = 6) hearts 4 weeks after TAC. y-axis indicates oxygen consumption rate (OCR) in pmol/min. **(E)** qPCR of mitochondrially encoded (mtDNA) genes *16S* and *ND1*, normalized to *HK2*, encoded by the nuclear genome (nuDNA). **(F)** *Sod2* mRNA levels measured by RNA-seq. **(G-H)** SOD2 immunoblot from heart lysates and quantification. Original immunoblot images are presented in Supplemental Fig. 9A.

Under normal conditions, the heart utilizes fatty acids as its main substrate for ATP generation. The failing heart increases glycolysis, without a concomitant increase in glucose oxidation, leading to overall decreased mitochondrial oxidative metabolism, resulting in an energy deficit^29^. Gene ontology analysis of the RNA-seq data revealed that WT TAC mice showed significant downregulation of cellular respiration and mitochondrial matrix composition pathways (Fig. 3E). Similarly, SIRT5OE mice post-TAC also showed reduced expression of genes in various catabolic processes, including fatty acid and small molecule metabolite breakdown (Fig. 3F). Comparison of the WT and SIRT5OE TAC transcriptomes revealed up-regulation of hallmark glycolytic genes in the WT TAC samples. Conversely, genes for fatty acid metabolism and components of the respiratory electron transport chain (ETC) were enriched in the SIRT5OE TAC samples (Fig. 4A). Overall, SIRT5OE TAC hearts exhibits an intermediate metabolic state between healthy (sham) hearts and the failing, WT TAC hearts 4 weeks after TAC.

To investigate whether RNA expression differences in these metabolic pathways manifested at the protein level, we immunoblotted for components of the ETC, and found similar reductions in expression in both TAC groups (Fig. 4B-C, Supplemental Fig. 3A). We then directly assessed ETC activity using mitochondria isolated from hearts four weeks post-TAC using the Agilent/Seahorse XFe96 analyzer. Both groups exhibited similar rates of election flow (Fig. 4D).

With comparable protein expression levels of ETC components, and electron flow, we then asked whether differences in total mitochondrial content might contribute to the discrepancies in LV function between the two TAC groups at four weeks post-surgery. Mitochondrial mass was quantified using two distinct approaches. First, qPCR was performed for mitochondrial protein-encoding genes *16S* and *Nd1,* and normalized to *Hk2.* Then, each sample was normalized to WT sham (Fig. 4E)^30^. Second, we assessed mRNA and protein levels of the mitochondrial protein SOD2 (Fig. 4F-H). In both assays, TAC resulted in a notable decrease in mitochondrial content, particularly in WT mice. However, only modest differences were evident between genotypes in the TAC groups. From these data, overall we conclude that while WT and SIRT5OE TAC hearts show transcriptional differences related to electron transport and other metabolic pathways, these expression differences do not robustly manifest at the level of electron transport chain activity, levels of respiratory complexes proteins, or mitochondrial content.

**Figure 5:**
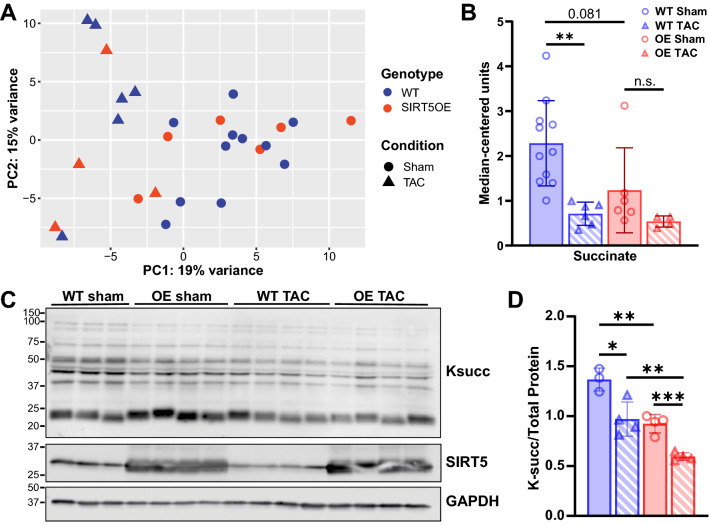
TAC alters the cardiac metabolomic landscape. **(A)** PCA of the metabolomics data from hearts four weeks post-surgery across all groups [WT sham (n = 10), WT TAC (*n* = 6), SIRT5OE sham (*n* = 6) and SIRT5OE TAC (*n* = 4)]. **(B)** Succinate levels. **(C-D)** Immunoblot analysis of Ksucc in sham and TAC mice, with quantification. Statistical significance was determined using two-way ANOVA followed by Sidak’s correction for multiple comparisons for 4-group analyses. Original immunoblot images are presented in Supplemental Fig. 10A.

To test whether the cardio-protective properties of SIRT5 overexpression might be associated with alterations in the metabolome, we performed unbiased metabolomics on cardiac samples across all groups at four weeks post-surgery using LC/MS-based mass spectrometry. PCA showed minimal variance between samples on the first two principal components (Fig. 5A). TAC was the main influence on sample clustering, with minimal contribution by genotype. Analysis of metabolite differences between genotypes also supported this conclusion, with only 6 and 4 metabolites significantly altered in WT sham to SIRT5OE sham and WT TAC to SIRT5OE TAC comparisons, respectively. In contrast, the WT sham to TAC comparison showed the greatest number of significantly different metabolites (44), followed by the SIRT5OE sham to TAC comparison (25).

Increased glucose usage and glycolytic flux are commonly observed during HF^31^. Levels of glycolytic metabolites did not show any significant changes between groups (Supplemental Fig. 4A-B). In contrast, several TCA cycle metabolites were significantly affected by TAC (Supplemental Table 2, Supplemental Fig. 4C-D). Succinate levels exhibited an interesting pattern, showing decreased levels with both TAC and SIRT5 overexpression (Fig. 5B). Since succinate is a substrate of SDH, a known SIRT5 target, we directly measured SDH activity but found no differences between the groups (Supplemental Fig. 5A). This result was consistent with electron flow data, where no differences in complex II/SDH activity were observed between mitochondria isolated from TAC hearts (Fig. 4C). Succinate is also linked to SIRT5 through SIRT5’s desuccinylase activity, as succinyl-CoA donates the succinyl group used to generate the succinyl-lysine modification (Supplemental Fig. 5B). Ksucc levels were lower in both SIRT5OE and TAC samples (Fig. 5C-D).

To complement these data, we obtained heart tissue samples from human HF patients. Similar to the mouse hearts, Ksucc levels trended lower in the human failing hearts compared to the control samples (*p* = 0.07) (Supplemental Fig. 5C-D). Therefore, cardiac stress leads to decreased levels of Ksucc, independent of *SIRT5* genotype, in both mice and humans. Taken together, transcriptomic and metabolomic analyses reflect a primarily TAC-driven, rather than genotype-associated, shift in the metabolic landscape in both genotypes following TAC.

### Cardiac fibrosis is suppressed in the SIRT5OE mice after TAC.

**Figure 6:**
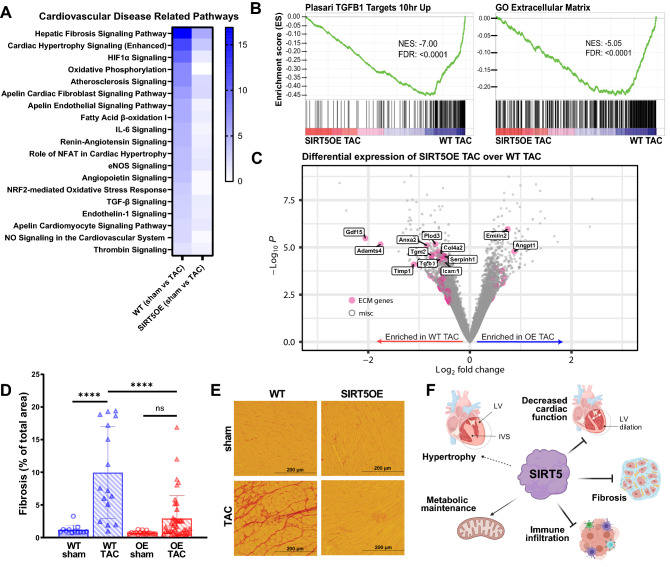
SIRT5OE protects against TAC-induced fibrosis four weeks after surgery. **(A)** Heat map of gene ontology analysis of WT (sham vs TAC) and SIRT5OE (sham vs TAC) mapping to cardiovascular disease related pathways using IPA. Blue color scale represents -log(p-value) of each pathway. **(B)** GSEA of SIRT5OE TAC compared to WT TAC RNA-seq samples. NES: normalized enrichment score. FDR: false discovery rate. **(C)** Volcano plot highlighting significant genes in from the GO: collagen containing extracellular matrix gene list. **(D)** Quantification of the amount of fibrosis in each heart sample, expressed in percentage [WT sham (*n* = 3), WT TAC (*n* = 4), SIRT5OE sham (*n* = 4) and SIRT5OE TAC (*n* = 10)]. Two-way ANOVA interaction term: p-value = 0.0012. **(E)** Representative images of heart sections stained with Picrosirius red for collagen. **(F)** Summary figure of effects of SIRT5 overexpression during pressure overload.

To further investigate the underpinnings of the improved response of SIRT5OE mice to TAC, we extracted a list of pathways IPA categorized as “cardiovascular disease-related pathways” (Fig. 6A). These pathways play critical roles in the development and progression of pathologic hypertrophy and HF, and all were more significantly enriched in WT mice with TAC compared to SIRT5OE mice. Fibrosis-related pathways (*i.e.,* “Hepatic fibrosis signaling pathway”, “Apelin cardiac fibroblast signaling pathway”, “TGF-β signaling”) were readily apparent (Figs. 6A and 3E-G). Hepatic Fibrosis Signaling was the most significant pathway identified in the IPA analysis, and includes signaling occurring during fibroblast activation, an essential process during adaptive hypertrophy by which resident fibroblasts differentiate into myofibroblasts (Fig. 6A). However, sustained myofibroblast activity results in excessive cardiac remodeling and is also a major contributor to LV dysfunction^32^.

GSEA confirmed that WT TAC samples were significantly enriched with genes upregulated by TGFβ1, a cytokine central to the fibrotic activation response (Fig. 6B). TGFβ1 triggers expression of a fibrotic gene program that stimulates proliferation, cell migration, and increased secretion various macromolecules that restructure the ECM and promote wound healing^33^. ECM-related genes were highly enriched in WT TAC samples, including ECM components (*Col1a1, Col4a2, Fbn1*), ECM modifying genes (*Timp1*, *Adamts4, Anxa2, Plod3*), and fibrotic signaling cytokines (*Tgfβ1, Gdf15, Icam1*) (Fig. 6B-C)^33,34^. mRNA levels of these genes were significantly lower in the SIRT5OE hearts after TAC. Thus, SIRT5 overexpression suppresses pressure overload-induced fibrosis signaling. These differences in mRNA expression were also reflected in tissue histology. Staining for collagen using picrosirius red showed a dramatic increase in ECM accumulation in WT TAC but not SIRT5OE TAC tissue (Fig. 6D-E). Thus, gene ontology and histologic analyses show that SIRT5 overexpression blunts TAC-induced cardiac fibrosis.

## Discussion

Taken together, these data demonstrate that SIRT5 is a limiting factor in maintaining cardiac structure and function during chronic pressure overload. Supra-physiological SIRT5 expression protects against HF-associated phenotypes, in particular systolic dysfunction and fibrosis (Fig. 6F). Elevated SIRT5 levels preserved cardiac contractile function after 4 weeks of pressure overload, at which point WT mice had developed systolic dysfunction, characterized by decreased EF, ventricular dilation, remodeling and fibrosis. Consistently, RNA-seq analysis revealed that key cardiac disease-related signaling pathways, including TGFβ, IL6, Renin-Angiotensin, and NFAT, were blunted in SIRT5OE TAC mice. These data demonstrate a cardioprotective role of SIRT5 in response to stress and are consistent with several previous studies showing that SIRT5KO mice are more susceptible to damage and dysfunction after cardiac stress^12,16–18^.

At least three non-mutually exclusive models may explain how SIRT5 confers protection against the deleterious consequences of TAC-induced pressure overload. First, SIRT5-mediated regulation of specific metabolic pathways is an attractive model, as metabolic dysfunction is both a driver and a consequence of hypertrophy and HF^29^. A variety of studies have shown that SIRT5-dependent regulation of its targets can be highly context-dependent^35^. Studies conducted using SIRT5KO mice and cell culture models have indicated that SIRT5 promotes fatty acid oxidation by desuccinylating ECHA^12^. The heart shifts from fatty acid oxidation to increased glucose consumption upon hypertrophy and failure^29^. In our studies, WT TAC mice exhibited transcriptional downregulation of genes involved in fatty acid catabolism and oxidative phosphorylation, with concurrent upregulation of glycolytic genes, compared to SIRT5OE TAC mice. However, these transcriptional signatures were not reflected in changes in ETC protein expression, electron flow, or total mitochondrial content.

In an ischemia–reperfusion model, SIRT5KOs exhibited a larger infarct size, and inhibition of SDH reduced this infarct size in both WT and SIRT5KO mice^16^. Furthermore, succinate injection is sufficient to induce cardiomyocyte hypertrophy^36^. Our metabolomics analysis revealed a borderline significant elevation (*p* = 0.08) in succinate levels in the WT sham hearts compared to SIRT5OE sham hearts (Fig. 5B). Although SIRT5 regulates SDH activity in a cell- and context-dependent fashion^8,35,37^, we were unable to detect changes in SDH activity in our SIRT5OE mice. Nevertheless, overexpression of SIRT5 might protect against hypertrophy and HF through regulating levels of succinate, and/or other key metabolites.

ROS management may represent another target of SIRT5 in the context of SIRT5-mediated cardioprotection. Increased ROS production due to pathological stimuli, including cytokine signaling or mechanical stretching, can stimulate cardiomyocyte hypertrophy, ventricular dilation, and contractile dysfunction^38^. Rat myoblasts treated with H_2_O_2_ show decreased SIRT5 levels and induction of apoptosis; in contrast, we did not observe changes in SIRT5 transcript or protein levels after TAC (Supplemental Fig. 2A-B). In rat myoblasts, SIRT5 overexpression protects against H_2_O_2_-driven apoptosis through interactions with BCL-XL, an anti-apoptotic member of the Bcl-2 family^39^. In other cell types, SIRT5 reduces cellular ROS levels via desuccinylation-mediated activation of SOD1 and promotes NADPH production by desuccinylation and deglutarylation of IDH2 and G6PD, respectively^35^. Elevated cellular ROS induces expression of a specific set of genes to detoxify ROS, an effect mediated in part through the NRF2 transcription factor^40^. NRF2-mediated oxidative stress response was significantly enriched in both WT and SIRT5OE RNA-seq comparisons, but blunted in the latter group (Fig. 6A). Future studies are needed to determine whether this effect is a driver or a consequence of attenuated cardiac dysfunction in SIRT5OE mice.

Second, immune-related pathways were among the most significantly altered by SIRT5OE in the context of TAC (Fig. 3F). Early immune infiltration after TAC is necessary for the transition from ventricular hypertrophy to dilation during pressure overload^41,42^. Blocking this inflammatory response alleviates late LV remodeling and dysfunction, cardiac fibrosis, and T cell expansion^43–45^. Similarly, in our studies, both genotypes developed significant hypertrophy after 4 weeks of TAC, but SIRT5OE mice were protected against subsequent ventricular dilation and dysfunction (Fig. 2C-H). Although current information on SIRT5 function in the immune system is incomplete, SIRT5 might protect against TAC by dampening the pro-inflammatory response in macrophages, where SIRT5 has been shown to desuccinylate and activate PKM2, blocking LPS-driven macrophage activation^46^. Likewise, malonylation of GAPDH promotes TNFα translation and macrophage activation, although SIRT5’s role in this process is uncharacterized^47^. Consistently, single-cell RNA-seq data from TAC mice reveals that SIRT5 is expressed in macrophage and T-cell populations, in addition to cardiomyocytes and other cell types in the heart (Supplemental Fig. 6A-B)^45^. In this regard, a pair of studies from the Hirschey group showed that while constitutive whole-body SIRT5KO mice displayed increased mortality after TAC, cardiomyocyte-specific SIRT5KOs and controls responded to TAC similarly, raising the possibility of cardiomyocyte-non-autonomous functions of SIRT5 in response to TAC^17,18^.

Third, SIRT5 might directly regulate cardiac fibrosis, a process initiated and sustained through a variety of signaling and mechanical factors, including inflammatory cytokines (*e.g.,* TGFβ, Angiotensin II, TNF*α*), neuroendocrine factors, and ventricular wall stretch^33^. These factors signal cardiac fibroblasts to activate, migrate, proliferate, and differentiate into myofibroblasts. In addition to ECM secretion and remodeling, activated fibroblasts exert protective effects through suppressing proinflammatory phenotypes and even promote the hypertrophic response through secretion of critical cytokines^48,49^. However, chronic myofibroblast activity inhibits oxygen and nutrient delivery, resulting in cardiomyocyte atrophy and death, ultimately leading to ventricular dilation and dysfunction^32,33^. The fibrotic response following TAC represents one of the most pronounced differences between WT and SIRT5OE mice. SIRT5 may play a role in regulating cytokine-mediated activation of fibrosis, in immune cells where cytokines originate, in triggering fibroblast activation, and/or in myofibroblasts themselves. Further studies are needed to determine (1) how long SIRT5 overexpression protects mice from dilation and contractile failure after TAC and (2) in which cell and tissue type(s) SIRT5 functions to confer cardioprotection.

In summary, we have shown that increased expression of the deacylase SIRT5 is sufficient to confer improved maintenance of cardiac function and decreased fibrosis during pressure overload. Sirtuin activity is amenable to enhancement by allosteric activators and NAD + precursors^35^. Intriguingly, we found that NADH levels and NAD^+^/NADH ratios were impacted by SIRT5 overexpression, those most of these differences failed to achieve statistical significance (Supplemental Fig. 4E, Supplemental Table 2)^50,51^. Overall, our results hint that SIRT5 might represent an attractive novel target in cardiac fibrosis, a condition that afflicts millions in the US and worldwide, but which currently lacks mechanism-based therapies.

## Methods

### Generation of SIRT5OE mice

Mouse ES cell clone 5-11C was derived from V6.5 ES cells^52^. ES cells were cultured in high-glucose Dulbecco’s minimal essential medium supplemented with 15% fetal bovine serum, 1 μM 2-mercaptoethanol, 4 mM glutamine, 50 IU of penicillin per ml, 50 ug of streptomycin per ml, and 1,000 U of recombinant leukemia inhibitory factor (ESGRO; Millipore) on mitotically inactivated feeder cells as described^53^. Chromosome counting indicated that 90% of twenty counted chromosome spreads contained an euploid chromosome number. ES cells were microinjected into 96 blastocysts obtained from the mating of albino mice (C57BL/6BrdCrHsd-*Tyr*^*c*^, Envigo). Of twenty-one pups born, twenty were ES cell-mouse chimeras and eighteen of these were male chimeras with at least 70% contribution from 5-11C ES cells, as judged by coat color contribution. Sox2-Cre (JAX 008,454) were mated with mice containing the SIRT5OE cassette to delete the loxP-STOP-loxP site to generate a global SIRT5OE transgenic mouse.

### Immunoblotting

Mice were anesthetized with isoflurane and immediately sacrificed by cervical dislocation. Heart tissue samples were flash frozen, pulverized, and resuspended in Laemmli sample butter (62.5 mM Tris pH6.8, 2% SDS, 10% glycerol, 5% β-mercaptoethanol, 1% bromophenol blue). Samples were sonicated for 30 s on ice, then spun down at 4 °C for 15 min at 15,000xg, and the supernatant was collected for protein quantification using a DC Protein Assay (Bio-Rad #5,000,112). Samples were boiled, resolved by SDS-PAGE, and transferred to PVDF membrane overnight at 4 °C using a Bio-Rad Criterion system. Membranes were stained with Ponceau S to assess protein loading, and then blocked using 5% milk in TBST (TBS containing 0.1% Tween-20) at room temperature. Primary antibodies were diluted in 5% BSA in TBST and incubated with the blot overnight at 4 °C on a rocker. Secondary incubation was performed at room temperature for 1 h using either mouse or rabbit secondary antibodies (Jackson ImmunoResearch 115–035-062 or 111–035-045) diluted 1:10,000 in 5% milk in TBST. For detection, blots were immersed in a western chemiluminescent HRP substrate (Millipore P90720) and imaged using the GE ImageQuant LAS 4000. Quantification was performed using ImageJ. Primary antibodies used in this study are listed in the Supplemental Methods.

### TAC and echocardiography

Detailed TAC and echocardiography procedures are detailed in the Supplemental Methods.

### Mouse Study approval

All mice were housed at the Biomedical Science Research Building (UM). All vertebrate animal experiments were approved by and performed in accordance with the regulations of the University of Michigan Institutional Animal Care & Use Committee (IACUC). All authors complied with ARRIVE guidelines and all methods are reported in accordance with ARRIVE guidelines.

### Human heart tissue procurement

Ventricular myocardial tissue from patients with advanced heart failure at the University of Michigan was collected at the time of cardiac transplantation following obtaining of written informed consent. Nonfailing ventricular myocardial tissue was collected from unmatched donor hearts from the University of Michigan. Co-morbidities in the donors that may have precluded use of their hearts for cardiac transplantation included age, hypertension, diabetes, minor coronary artery disease, alcohol or tobacco use. Prior to tissue retrieval, all hearts were perfused with ice-cold cardioplegia. Samples from each heart were snap frozen in liquid N2 at the time of arrival and stored at − 80 °C. This study was approved by the Institutional Review Boards of the University of Michigan Medical School (IRBMED) and performed in accordance with all relevant guidelines and regulations.

### Mitochondrial isolation

Mitochondrial isolation was performed as previously described^54^. In brief, mice were anesthetized with isoflurane and immediately sacrificed by cervical dislocation. After cardiac harvest, ventricles were minced in ice-cold MIB (210 mM D-Mannitol, 70 mM sucrose, 5 mM MgCl_2_, 10 mM KH_2_PO_4_, 5 mM HEPES, 1 mM EGTA, 0.2% fatty acid-free BSA), mechanically homogenized using a Fisher Scientific PowerGen 125, and centrifuged at 800×*g* then 8000×*g* for 10 min at 4 °C. The final pellets, containing mitochondria, were resuspended in MAS (220 mM D-Mannitol, 70 mM sucrose, 5 mM MgCl_2_, 10 mM KH_2_PO_4_, 2 mM HEPES, 1 mM EGTA, 0.1% fatty acid-free BSA). Mitochondria were quantified using DC quantification assy. Both MIB and MAS pH were adjusted to 7.2 with KOH and stored at 4 °C before use.

### Agilent/Seahorse assay

To assess the functionality of isolated mitochondria from WT and SIRT5OE hearts, the electron flow assay was performed using the XFe96 Extracellular Flux Analyzer (Agilent Technologies, Santa Clara, CA) per the manufacturer’s instructions. Specific methods used in this study are in Supplemental Methods.

### Metabolomics sample preparation

The metabolomics MS/MS system, liquid chromatography, and mass spectrometry protocols used are detailed in the Supplemental Methods. Mice were anesthetized with isoflurane and immediately sacrificed by cervical dislocation. Heart tissue samples were immediately flash frozen, pulverized to powder, vortexed, and centrifuged for 10 min at 13,000 × g, 4 °C. Then, 1 ml of the supernatant was aspirated from each tube, transferred to a tightly capped sample tube, and stored at − 80 °C until analysis.

### Metabolomics data analysis

The QqQ data were pre-processed with Agilent MassHunter Workstation QqQ Quantitative Analysis Software (B0700). Each metabolite abundance level in each sample was divided by the median of all abundance levels across all samples for proper comparisons, statistical analyses, and visualizations among metabolites. A total of 230 metabolites were measured. The statistical significance test was done by a two-tailed *t*-test with a significance threshold level of 0.05.

### SDH activity assay

Succinate dehydrogenase activity was measured from flash frozen and powderized sham and TAC hearts using a kit (ab228560) according to manufacturer’s instructions. Around 20 mg of heart was diluted in assay buffer at 1:20 ratio, homogenized using a dounce homogenizer, and 2.5 mg final, diluted tissue was used in the assay.

### Tissue preparation for RNA extraction

Heart tissue samples were flash frozen, pulverized, resuspended in TRIzol and homogenized using a power homogenizer (Fisher Scientific PowerGen 125). RNA was extracted using RNeasy Mini Kit (Qiagen 741–4) per the manufacturer’s instructions.

### qRT-PCR

cDNA was generated from RNA using SuperScript III Reverse Transcriptase (Invitrogen 18,080,044) per the manufacturer’s instructions. TaqMan Gene Expression Assays (Applied Biosystems) for single-tube assays were used for all qRT-PCR experiments. Reactions were carried out as per the manufacturer’s methods, using TaqMan Gene Expression Assays as listed with the associated TaqMan Assay ID: *Myh6* (Mm00440359_m1), *Acta1* (Mm00808218_g1), *Nppa* (Mm01255747_g1), *Myh7* (Mm00600555_m1).

### RNA-sequencing bioinformatic analysis

RNA libraries were prepared using a standard Illumina protocol. Base-calling was performed on an Illumina HiSeq4000. Raw fastq files were analyzed using FastQC for quality control. Transcripts were aligned to mm ensemble cDNA release 101 using kallisto (v0.46.0) and counted using Tximport (v1.18.0)^55–57^. DESeq2 was used for calculating differential expression^58^. Genes with a false discovery rate (FDR) of less than 0.05 and a log2-fold change greater that |1| were designated as differentially expressed between groups. Pathway analysis was performed using Ingenuity Pathway Analysis (IPA) and gene set enrichment analysis (GSEA)^27,28^.

### Cardiomyocyte size quantification

Sections were deparaffinized in xylene for three washes for 5 min each and rehydrated in 100% ethanol, then two 10-min washes each with 95% ethanol, followed by two 5-min washes in PBS, and staining with 10 μg/mL wheat germ agglutinin conjugated to Alexa Flour 488 (Invitrogen) diluted in PBS for one hour at room temperature. Sections were washed 3× with PBS, mounted with Prolong Gold with DAPI (Invitrogen), and imaged on Zeiss Confocal at 20× magnification. Three images of the left ventricle were taken of each cardiac section and cell surface area quantified on ImageJ from 200 cells per sample.

### Fibrosis staining

Heart tissue was paraffin embedded, sectioned, and stained with picrosirius red at the University of Michigan Dentistry Histology Core. Paraffin embedded heart section were de-paraffinized in xylene, rehydrated in 100% EtOH, 95% EtOH, and 70% EtOH and dH20. Sections were then fixed in 10% buffered formalin and stained in picric acid solution with 0.1% Sirius red, dehydrated in 80% EtOH, 95% EtOH, then 100% EtOH, cleared in xylene, and finally mounted with Permount. For each heart, eight images of left ventricle sections, 500 μm × 400 μm, were acquired under polarized bright-filed light for fibrosis detection. Collagen area fraction (CAF) of the total areas, 500 μm × 400 μm, were measured by ImageJ using the equation ((collagen area)/(total area) = CAF). 4 images with the highest CAF were used for statistical analysis.

### Statistics

Statistical analysis were conducted using Graphpad Prism, version 8. Multiple-group comparisons were analyzed using two-way ANOVA followed by Sidak’s correction for multiple comparisons. For analysis of two groups, Welch’s t-test was used. A p-valu*e* of less than 0.05 was considered statistically significant. Scatter plots include median and interquartile range markers. Significance markers: (*) *p* < 0.05, (**) *p* < 0.01, (***) *p* < 0.001, (****) *p* < 0.0001.

## Supplementary Information

Supplementary Information 1.

Supplementary Information 2.

## Data Availability

Both the raw and analyzed data generated from this study are openly available at GEO (GSE198926).
