# Supplementary material for: Sirtuin 5 levels are limiting in preserving cardiac function and suppressing fibrosis in response to pressure overload
Source: Sci Rep. 2022 Jul 18;12:12258. doi: 10.1038/s41598-022-16506-7 (PMC9293976; doi:10.1038/s41598-022-16506-7)
Supplement: Supplementary file 1 — Supplementary Information 1. [file 41598_2022_16506_MOESM1_ESM.pdf]

Supplemental Figure 1

A

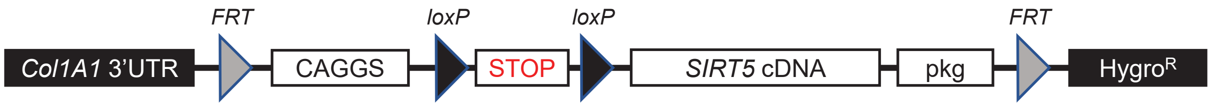

B

Parents: WT male, SIRT5OE female					χ^2	p-value
	WT	SIRT5OE	Total			
Males	73	77	150		2.09	0.55
Females	74	61	135			
Total	147	138	285			

Parents: SIRT5OE male, WT female					χ^2	p-value
	WT	OE	Total			
Males	38	25	63		5.07	0.17
Females	42	41	83			
Total	80	66	146			

Combined					χ^2	p-value
	WT	SIRT5OE	Total			
Males	111	102	213		1.32	0.72
Females	116	102	218			
Total	227	204	431			

Supplemental Figure 1. Generation and characterization of SIRT5OE mice. (A) Schematic of the transgenic SIRT5 overexpression cassette. A cassette containing a constitutive CAGGS promoter, a transcriptional flox-STOP-flox sequence, followed by the SIRT5 cDNA, was inserted into the collagen1 A1 (Col1A1) 3'UTR by FLP recombination. (B) Numbers of male and female mice born per WT or SIRTOE litter, grouped by parental genotype.

Supplemental Figure 2

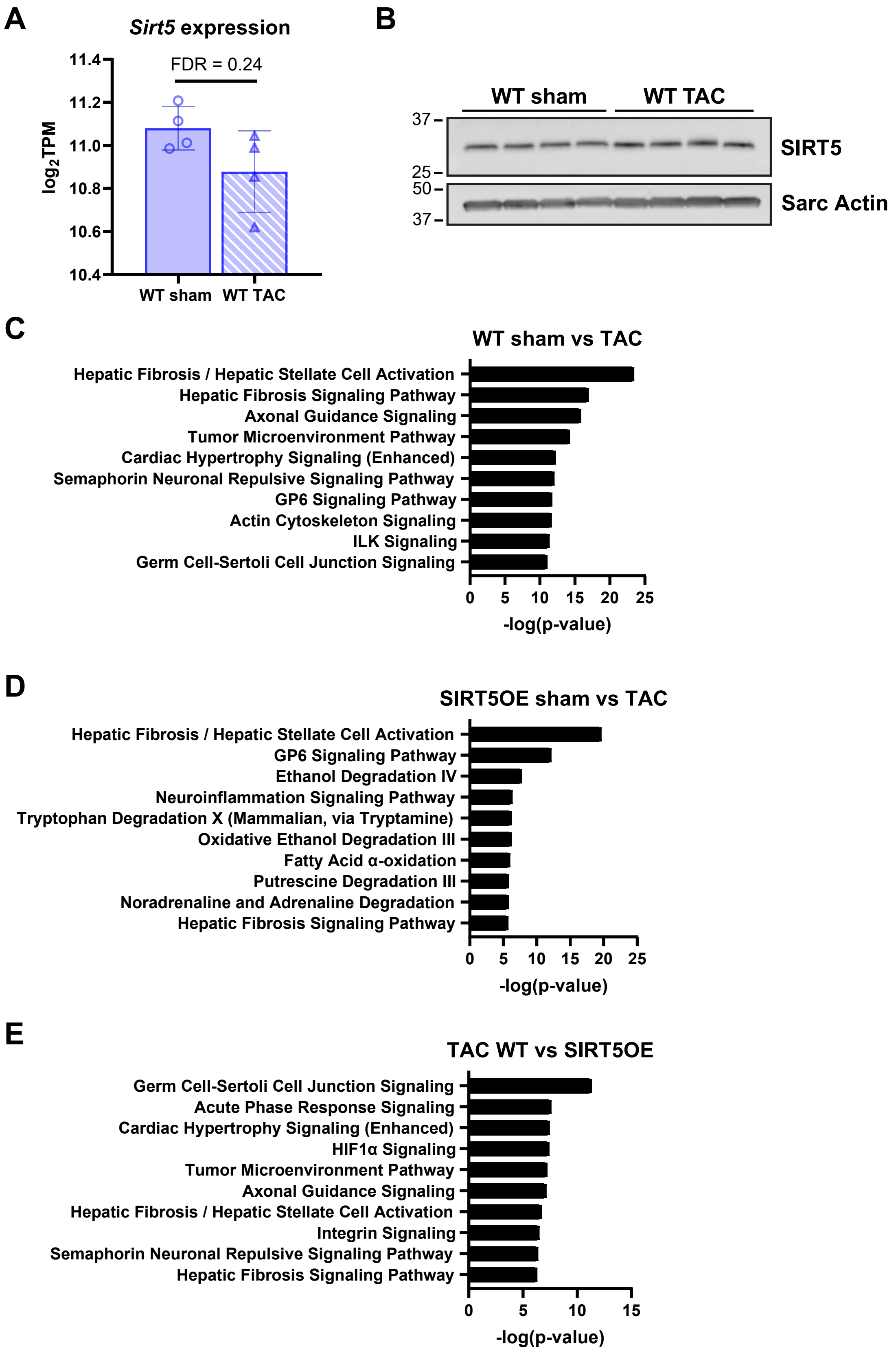

Supplemental Figure 2. RNA-seq gene ontology analysis. (A) Sirt5 expression in WT sham and WT TAC hearts by RNA-seq. (B) SIRT5 protein levels in WT sham and TAC heart lysates. Original immunoblot images are presented in Supplemental Figure 11a. (C-E) Top 10 GO pathways, sorted on significance,enriched by differentially expressed genes in each labelled comparison, determined by IPA.

Supplemental Figure 3

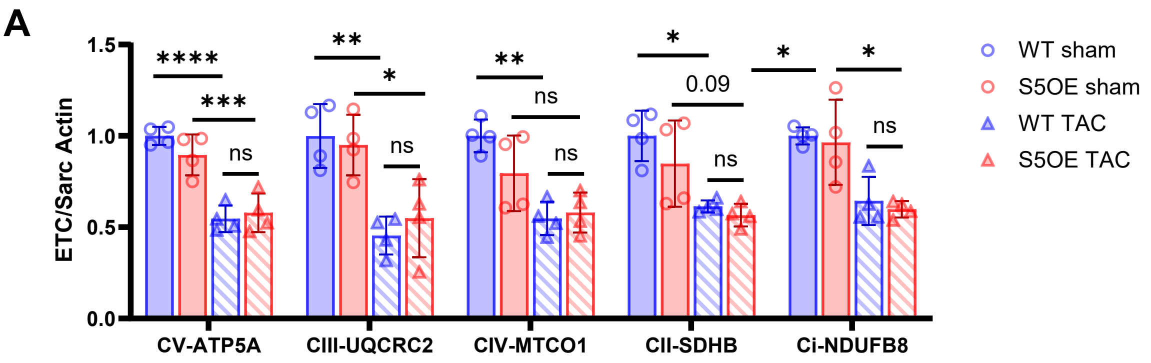

Supplemental Figure 3. Mitochondrial content analysis. (A) Quantification of each ETC protein in Figure 4B.

Supplemental Figure 4

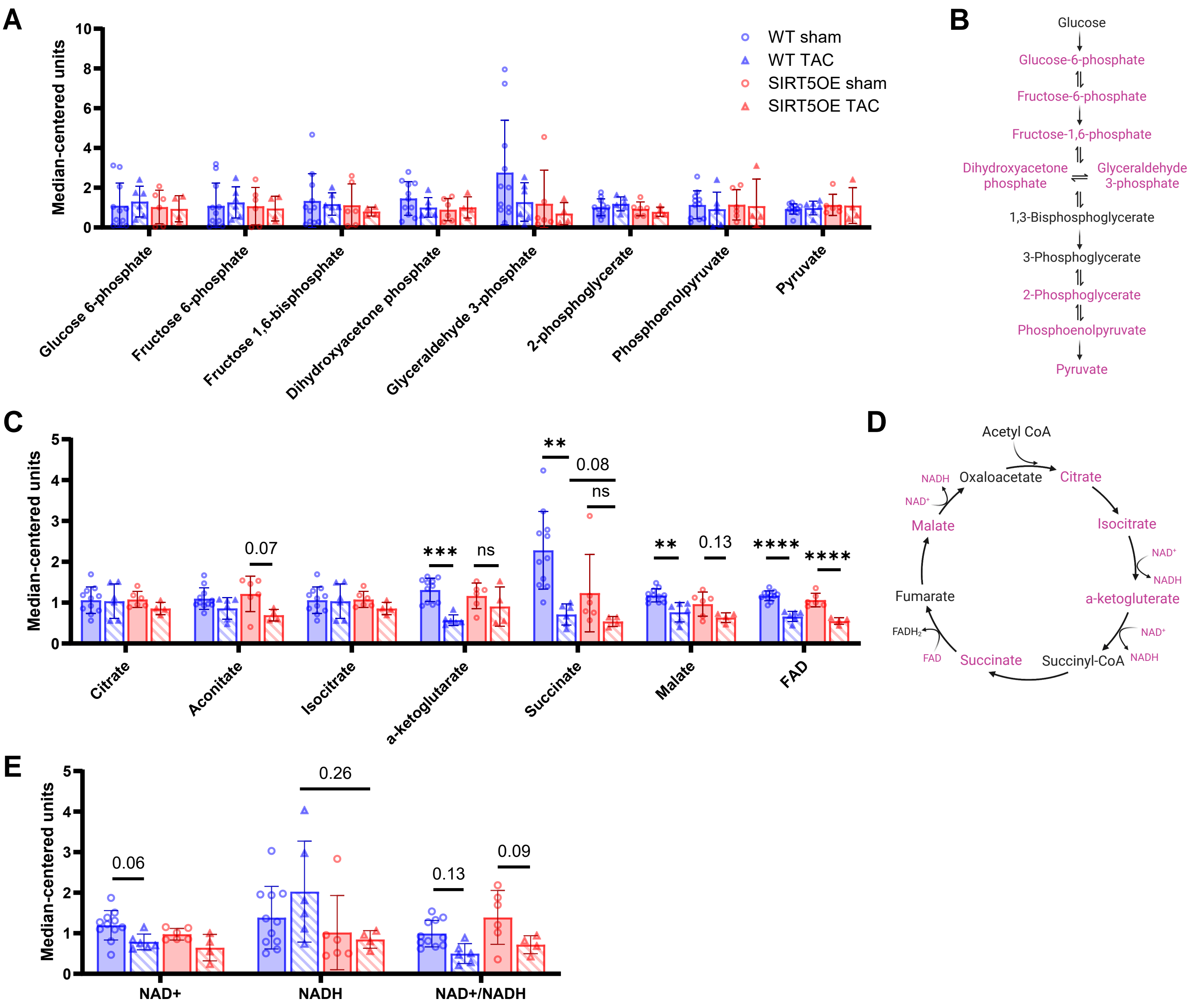

Supplemental Figure 4. Metabolites of key metabolic pathways. (A,C,E) Metabolites of glycolysis (A), the TCA cycle (C), and NAD+/NADH (E), plotted using median-centered values. (B,D) Cartoon schematics of glycolysis and the TCA cycle. Metabolites highlighted in magenta in the schematics represent measured metabolites.

Supplemental Figure 5

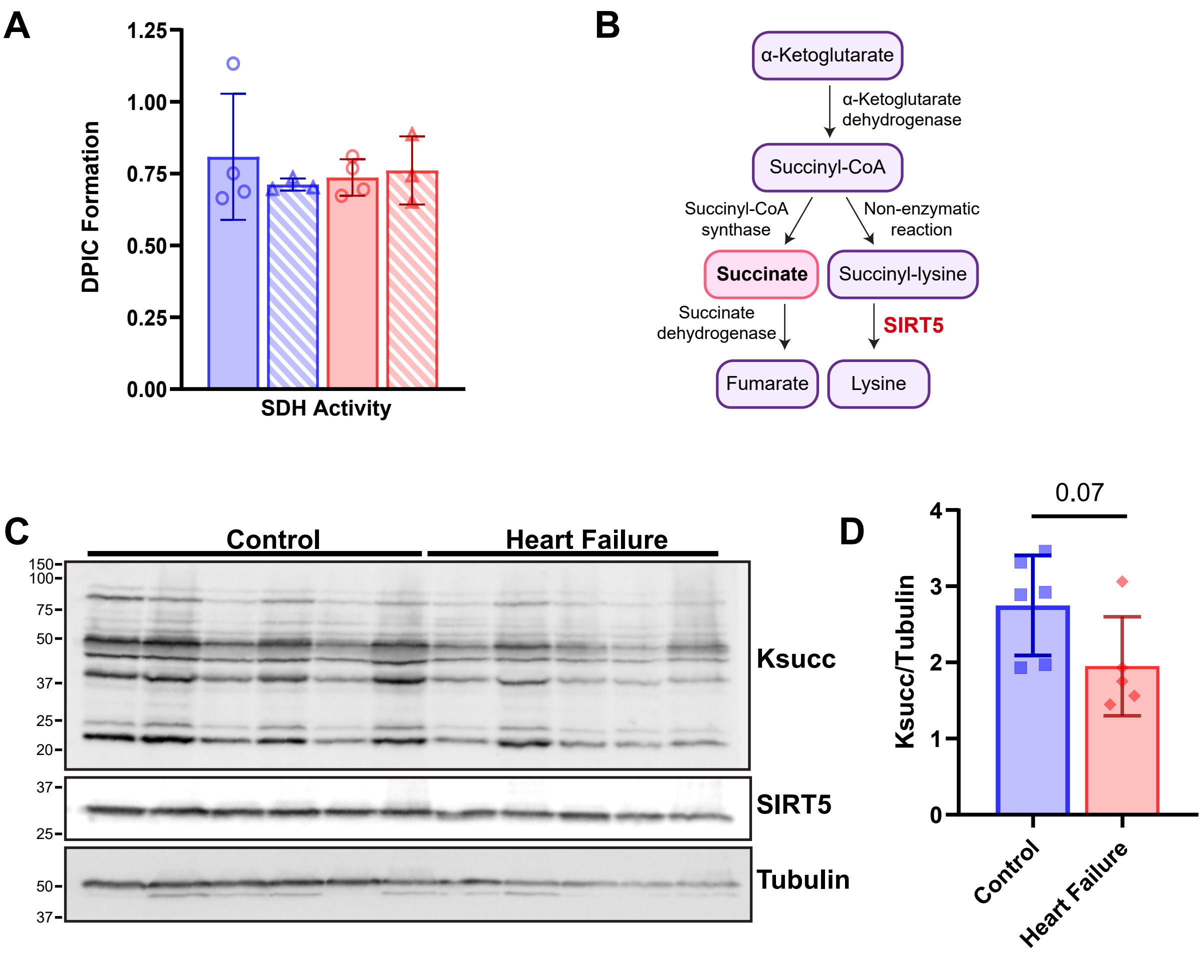

Supplemental Figure 5. TAC alters succinate and protein succinylation. (A) Cardiac SDH activity. (B) Schematic of succinate in relation to SIRT5. (C-D) Immunoblot analysis of Ksucc from human heart failure patients and controls with quantification. Original immunoblot images are presented in Supplemental Figure 11B.

Supplemental Figure 6

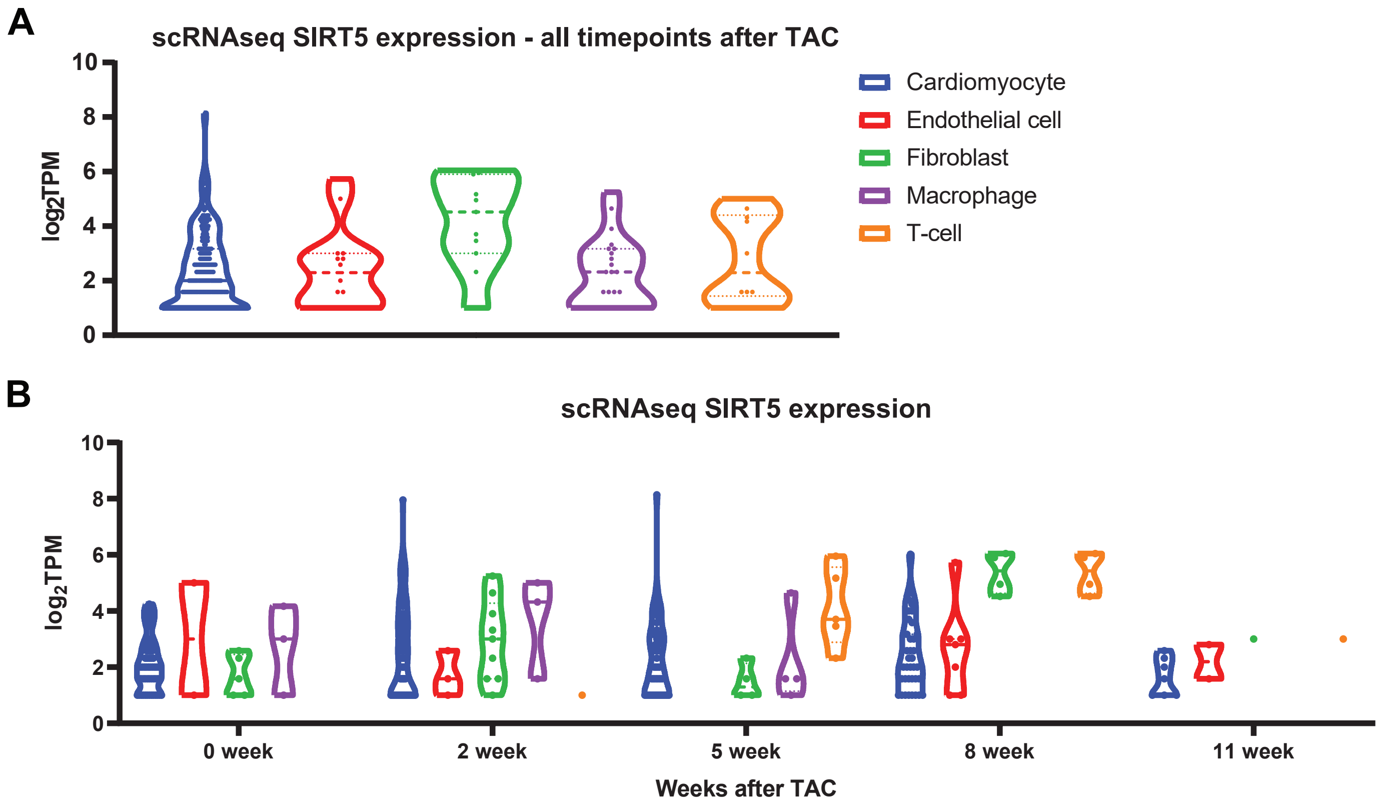

Supplemental Figure 6. SIRT5 expression in bulk heart tissue and specific cell populations. (A) Sirt5 expression in five major cell types using single-cell data generated by Ren *et al*[45]. Only cells with at least 2 reads of Sirt5 were plotted. (B) Sirt5 expression in single-cell RNA-seq across 5 different time-points through 11 weeks of pressure overload.

Supplemental Figure 7

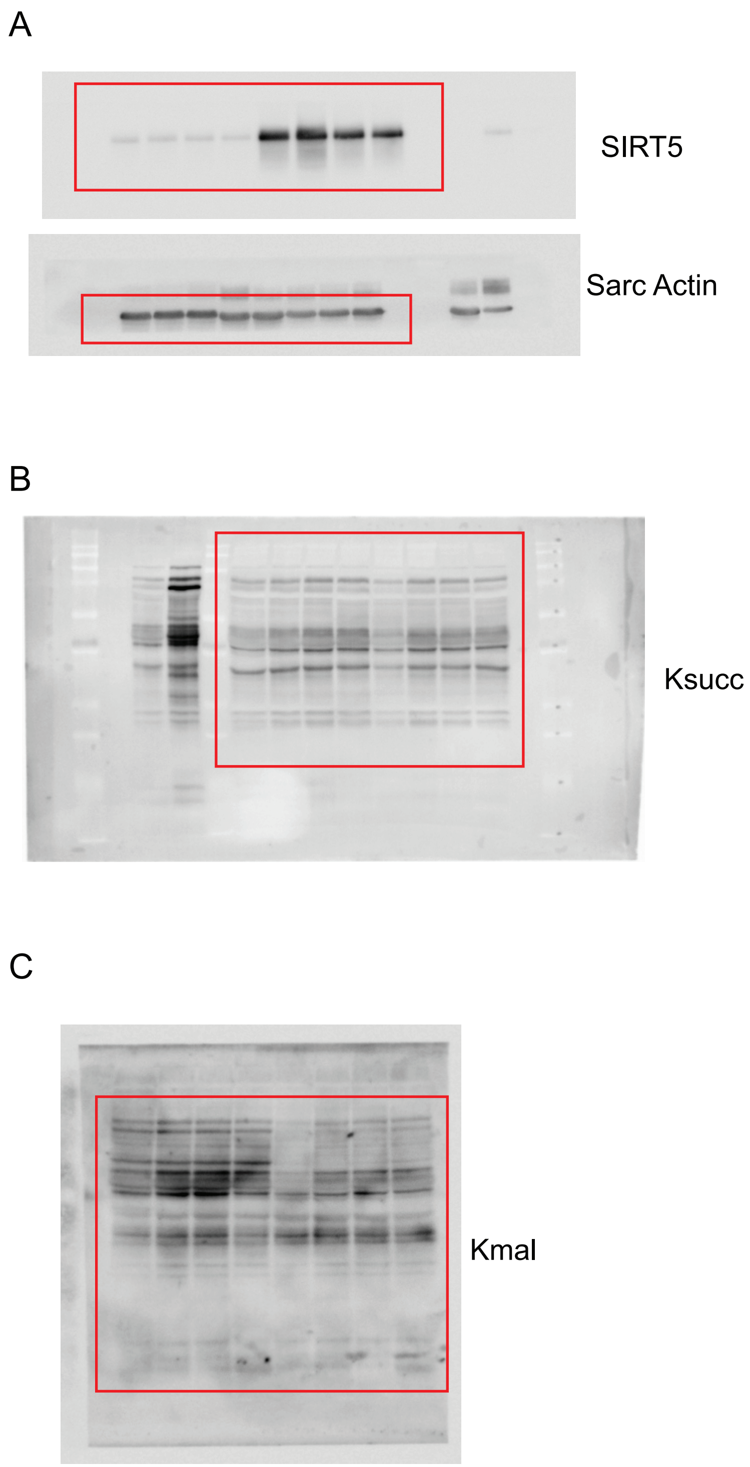

Supplemental Figure 7. Figure 1 original blots. (A) Figure 1A. (B) Figure 1C. (C) Figure 1D.

Supplemental Figure 8

A

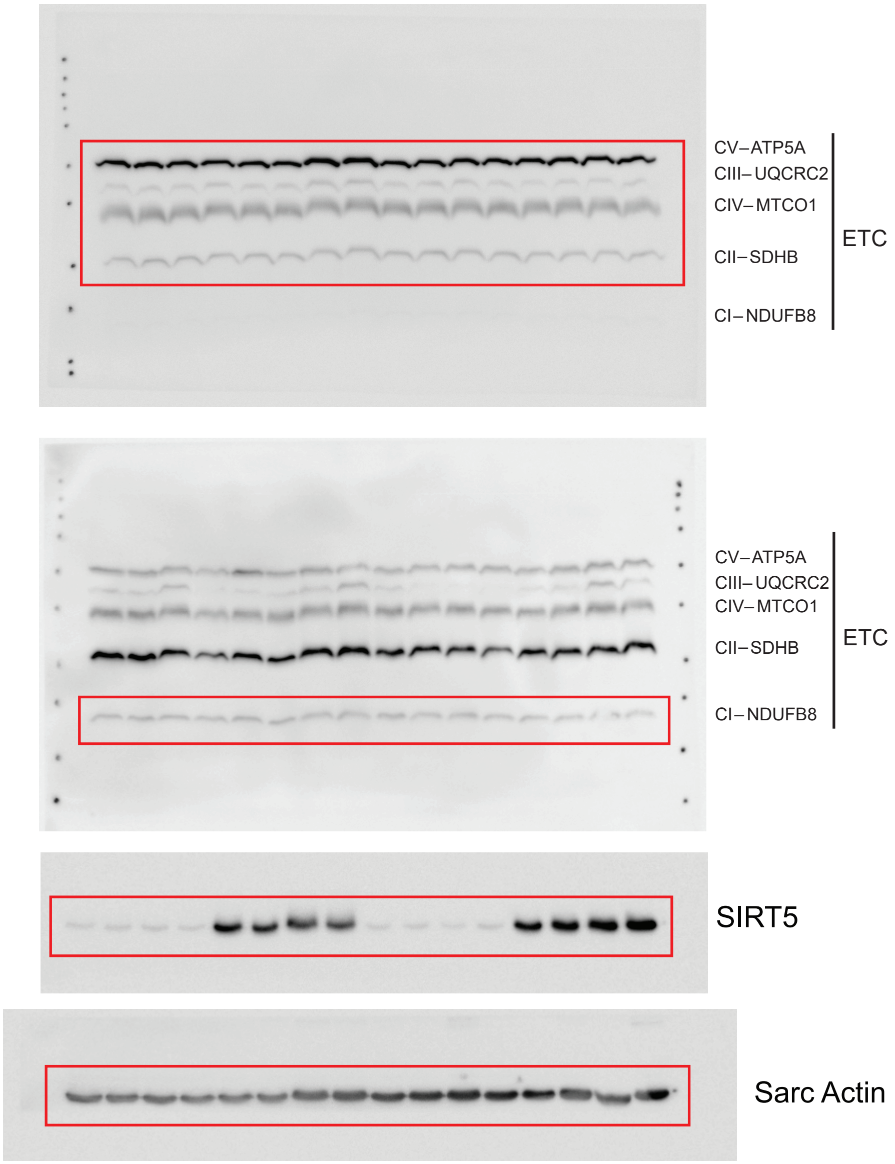

Supplemental Figure 8. Figure 4 original blots. (A) Figure 4B

Supplemental Figure 9

A

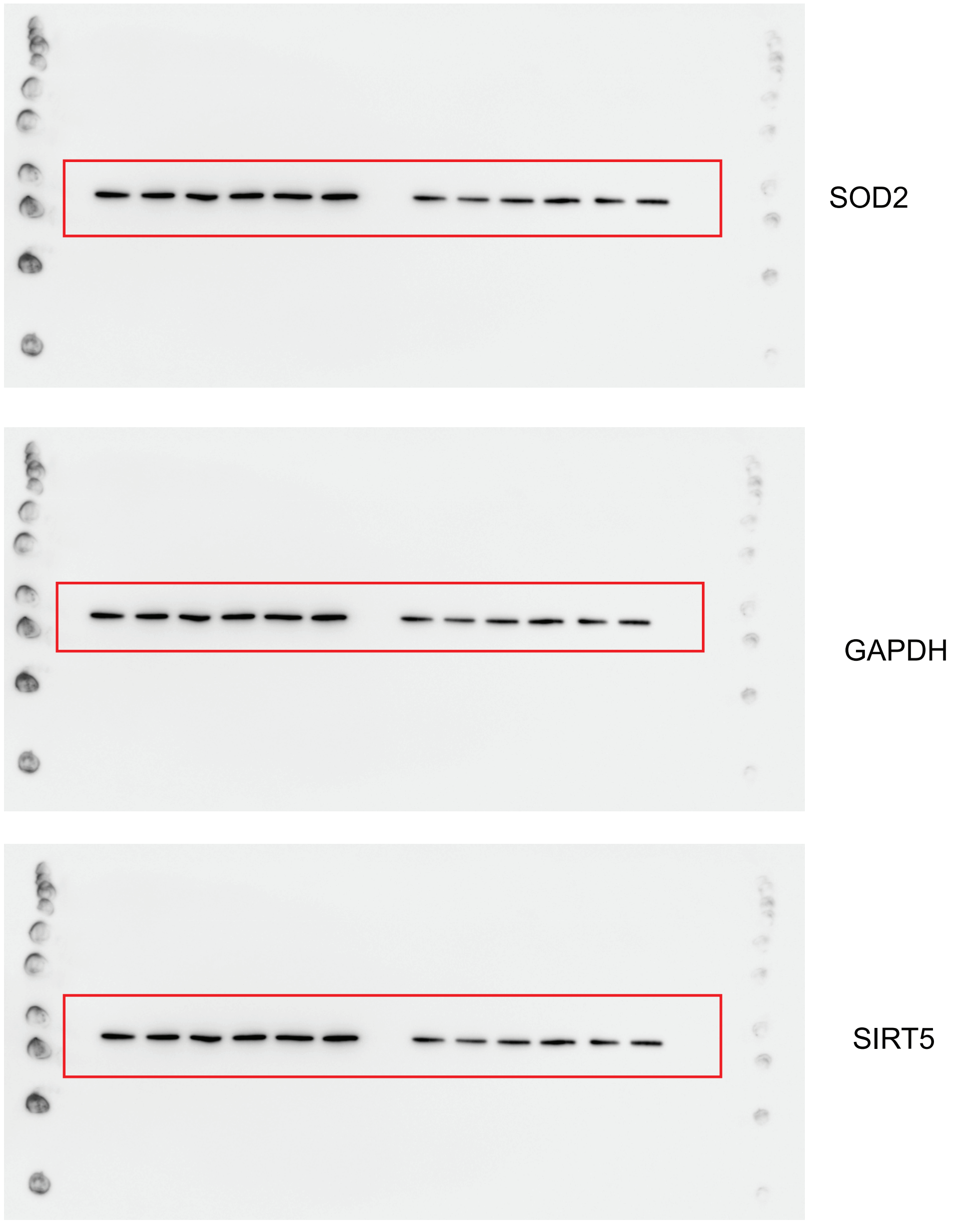

Supplemental Figure 9. Figure 4 original blots. (A) Figure 4G

Supplemental Figure 10

A

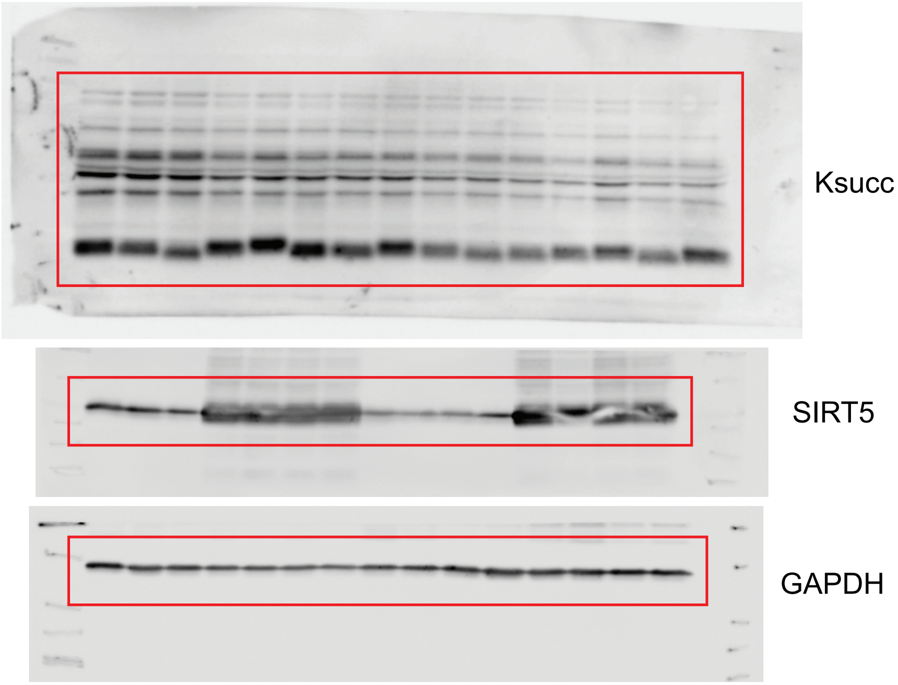

Supplemental Figure 10. Figure 5 original blots. (A) Figure 5C.

Supplemental Figure 11

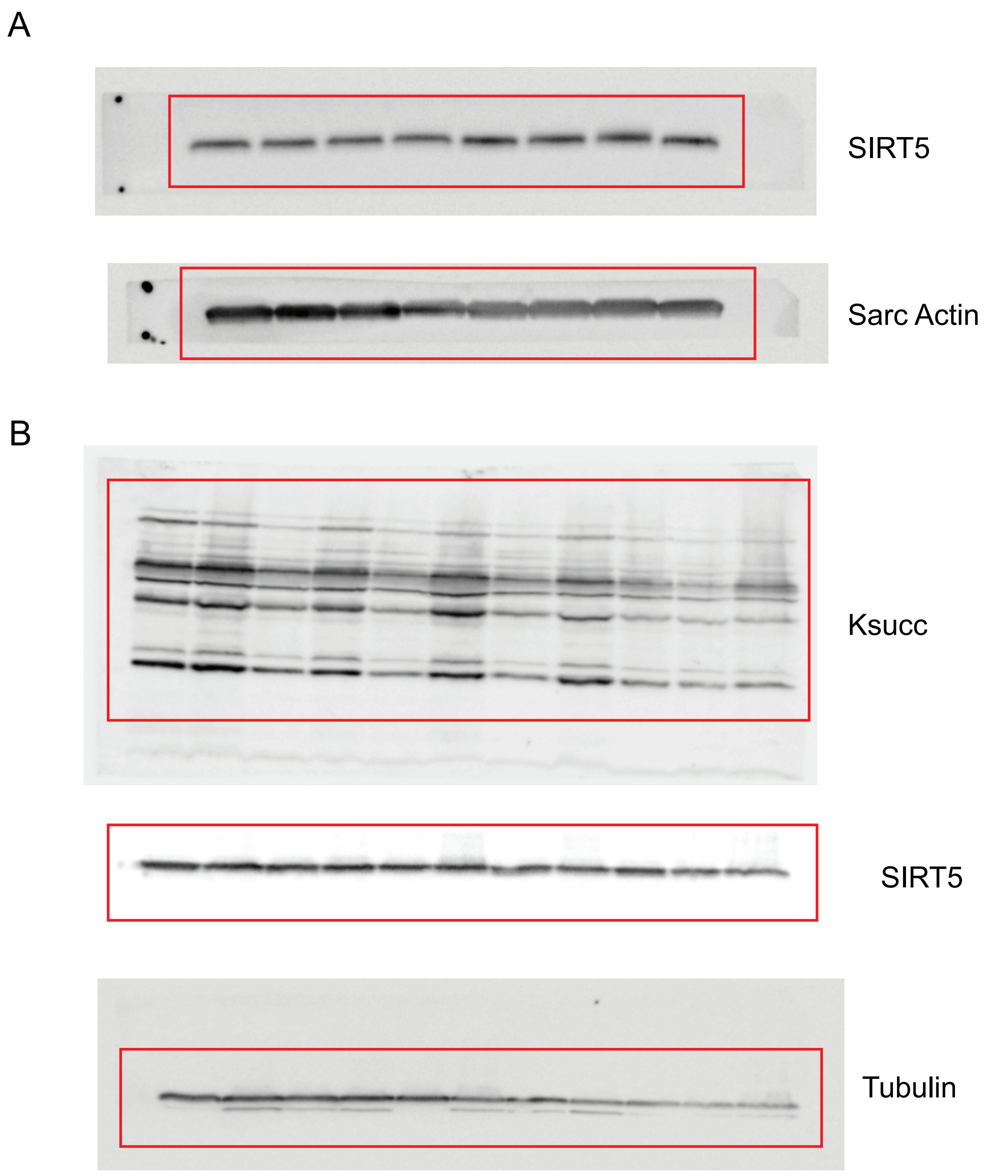

Supplemental Figure 11. Supplemental figure original blots. (A) Supplemental Figure 2B. (B) Supplemental Figure 5C.

Supplemental Table 1					
	Total			Died	
	WT TAC	S5OE TAC		WT TAC	S5OE TAC
Batch 1	8	12		2	2
Batch 2	7	5		1	1
Batch 3	4	4		0	1
**Total**	**19**	**21**		**3**	**4**

Supplemental Table 1. TAC-associated mortality versus genotype.

Supplemental Table 2			
	**Genotype Effect**	**TAC Effect**	**Interaction Effect**
**NAD^+**	0.136	0.005*	0.740
**NADH**	0.044*	0.522	0.275
**NAD^+/NADH**	0.074$^+$	0.002*	0.599
**Glucose 6-phosphate**	0.584	0.888	0.705
**Fructose 6-phosphate**	0.694	0.945	0.712
**Fructose 1,6-bisphosphate**	0.504	0.598	0.850
**Dihydroxyacetone phosphate**	0.326	0.555	0.321
**Glyceraldehyde 3-phosphate**	0.197	0.235	0.548
**2-phosphoglycerate**	0.133	0.940	0.306
**Phosphoenolpyruvate**	0.825	0.696	0.832
**Pyruvate**	0.399	0.962	0.826
**Citrate/Isocitrate**	0.532	0.345	0.441
**Aconitate**	0.840	0.005*	0.263
**a-ketoglutarate**	0.460	<0.001*	0.065$^+$
**Succinate**	0.069$^+$	0.002*	0.183
**Malate**	0.058$^+$	<0.001*	0.638
**FAD**	0.049*	<0.001*	0.940

Supplemental Table 2. Two-way ANOVA analyses results of all metabolites plotted in Supplemental figure 4. Statistical significance was determined using two-way ANOVA. Significance markers: (*) $p < 0.05$, (+) $p < 0.1$.
