# Supplementary material for: Sirtuin 5 levels are limiting in preserving cardiac function and suppressing fibrosis in response to pressure overload
Source: Sci Rep. 2022 Jul 18;12:12258. doi: 10.1038/s41598-022-16506-7 (PMC9293976; doi:10.1038/s41598-022-16506-7)
Supplement: Supplementary file 2 — Supplementary Information 2. [file 41598_2022_16506_MOESM2_ESM.docx]

**Supplemental Methods**

*Antibodies used in this study*: SIRT5 (Cell Signaling #8782), Succinyl-lysine (PTMBiolabs #PTM-419), Malonyl-lysine (PTMBiolabs #PTM-901), SOD2 (Santa Cruz #30080), GAPDH (Cell Signaling #5174P), Rodent OXPHOS (Abcam #ab110413), Sarcomeric Actin (Sigma # A2172-100UL), Tubulin (Santa Cruz # SC-23948).

*TAC procedure:* Carprofen use administered pre-emptively and for 48 hours, then as needed. The animals were intubated and ventilated. The aortic arch was isolated by entering the extrapleural space above the first rib, and the transverse aorta was isolated between the right and left carotid arteries. A 7-0 or similar size nylon suture ligature was tied around the transverse aorta against a 27-gauge needle to produce a 60-70% constriction after the removal of the needle. The incision was closed, and the left-sided pneumothorax (if present) was evacuated.

*Echocardiography:* Induction of anesthesia was performed in an enclosed container filled with 5% isoflurane. After induction, the mice were placed on a warming pad to maintain body temperature. 1 – 1.5% isoflurane was supplied via a nose cone. Hair was removed from the upper abdominal and thoracic area with depilatory cream. ECG was monitored via non-invasive **resting ECG electrodes. Transthoracic echocardiography was performed in the supine or left lateral position. Two-dimensional, M-mode, Doppler and tissue Doppler echocardiographic images were recorded using** a Visual Sonics’ Vevo 2100 high resolution *in vivo* micro-imaging system. LV ejection fraction was measured from the two-dimensional long axis view. Systolic and diastolic dimensions and wall thickness were measured by M-mode in the parasternal short axis view at the level of the papillary muscles. Fractional shortening and ejection fraction were also calculated from the M-mode parasternal short axis view. Diastolic function was assessed by conventional pulsed-wave spectral Doppler analysis of mitral valve inflow patterns (early [E] and late [A] filling waves). Doppler tissue imaging (DTI) was used to measure the early (Ea) diastolic tissue velocities of the septal and lateral annuluses of the mitral valve in the apical 4-chamber view.

*Agilent Seahorse Assay:* Briefly, 1μg of isolated mitochondria (from either WT or SIRT5OE mice) in 25μl of mitochondrial assay solution (MAS), supplemented with 10mM pyruvate, 2mM malate, and 4μM FCCP, were plated per well of a 96-well Seahorse microplate on ice. The microplate was then transferred to a centrifuge equipped with a swinging bucket microplate adaptor, and spun at 2000g for 20 minutes at 4°C. After centrifugation, 155μL of pre-warmed (37°C) MAS, supplemented with 10mM pyruvate, 2mM malate, and 4μM FCCP, was added to each well. Mitochondrial oxygen consumption rates (OCR) were then measured, under initial conditions and following addition of 2μM rotenone (Sigma #R8875), 10mM succinate (Sigma #S9515), 4 μM antimycin A (Sigma #A8674), and 10mM ascorbate (Sigma #A7506) plus 100μM TMPD (Sigma #T3134).

*Metabolomics MS/MS system:* Samples were run on an Agilent 1290 Infinity II LC-6470 Triple Quadrupole (QqQ) tandem mass spectrometer (MS/MS) system with the following parameters: Agilent Technologies Triple Quad 6470 LC-MS/MS system consists of the 1290 Infinity II LC Flexible Pump (Quaternary Pump), the 1290 Infinity II Multisampler, the 1290 Infinity II Multicolumn Thermostat with 6 port valve and the 6470 triple quad mass spectrometer. Agilent Masshunter Workstation Software LC/MS Data Acquisition for 6400 Series Triple Quadrupole MS with Version B.08.02 was used for compound optimization, calibration, and data acquisition.

*Metabolomics liquid chromatography*: 2μLof sample was injected into an Agilent ZORBAX RRHD Extend-C18 column (2.1 × 150 mm, 1.8 um) with ZORBAX Extend Fast Guards. The LC gradient profile is as follows, solvent conditions below. 0.25 ml/min, 0-2.5 min, 100% A; 2.5-7.5 min, 80% A and 20% B; 7.5min-13 min 55% A and 45% B; 13min-24 min, 1% A and 99% B; 24min-27min, 1% A and 99% C; 27min-27.5min, 1% A and 99% C; at 0.8 ml/min, 27.5-31.5 min, 1% A and 99% C; at 0.6 ml/min, 31.5-32.25min, 1% A and 99% C; at 0.4 ml/min, 32.25-39.9 min, 100% A; at 0.25 ml/min, 40 min, 100% A. Column temp is kept at 35 ̊C, samples are at 4 ̊C. *LC Solvents*: Solvent A is 97% water and 3% methanol 15 mM acetic acid and 10 mM tributylamine at pH of 5. Solvent B is 15 mM acetic acid and 10 mM tributylamine in methanol. Washing Solvent C is acetonitrile. LC system seal washing solvent 90% water and 10% isopropanol, needle wash solvent 75% methanol, 25% water. Solvents were purchased from the following vendors: GC-grade Tributylamine 99% (ACROS ORGANICS), LC/MS grade acetic acid Optima (Fisher Chemical), InfinityLab Deactivator additive, ESI –L Low concentration Tuning mix (Agilent Technologies), LC-MS grade solvents of water, and acetonitrile, methanol (Millipore), isopropanol (Fisher Chemical).

*Metabolomics mass spectrometry*: 6470 Triple Quad MS is calibrated with the Agilent ESI-L Low concentration Tuning mix. Source parameters: Gas temp 150 ̊C, Gas flow 10 l/min, Nebulizer 45 psi, Sheath gas temp 325 ̊C, Sheath gas flow 12 l/min, Capillary -2000 V, Delta EMV -200 V. Dynamic MRM scan type is used with 0.07 min peak width, acquisition time is 24 min. Delta retention time of plus and minus 1 min, fragmentor of 40 eV and cell accelerator of 5 eV are incorporated in the method. The MassHunter Metabolomics Dynamic MRM Database and Method was used for target identification. Key parameters of AJS ESI were: Gas Temp: 150 ̊C, Gas Flow 13 l/min, Nebulizer 45 psi, Sheath Gas Temp 325 ̊C, Sheath Gas Flow 12 l/min, Capillary 2000 V, Nozzle 500 V. Detector Delta EMV(-) 200.
